# Effect of surface modification on silica supported Ti catalysts for cyclohexene oxidation with vapor-phase hydrogen peroxide†

**DOI:** 10.1039/d4ra04552a

**Published:** 2024-08-13

**Authors:** Sol Ahn, Sarah K. Friedman, Justin M. Notestein

**Affiliations:** a Department of Chemical Engineering, Chung-Ang University 84 Heukseok-ro, Dongjak-gu Seoul 06974 Republic of Korea solahn@cau.ac.kr; b Department of Chemical and Biological Engineering USA j-notestein@northwestern.edu; c Center for Catalysis and Surface Science 2145 Sheridan Rd Evanston Illinois 60208 USA

## Abstract

Surface modification *via* grafting of organic moieties on a Lewis acid catalyst (silica supported Ti catalyst, Ti-SiO_2_) alters the activation of H_2_O_2_ in vapor-phase cyclohexene epoxidation. Grafting a fluorous group (1*H*,1*H*-perfluoro-octyl) suppresses activity of Ti-SiO_2_. Conversely, grafting either a nonpolar group (octyl) or a polar aprotic group (triethylene glycol monomethyl ether) enhances rates and shifts selectivity toward *trans*-1,2-cyclohexanediol.

## Abstract 2

A methodology of modifying surface properties of a Lewis acid catalyst for cyclohexene oxidation with vapor-phase H_2_O_2_.

## Graphical abstract

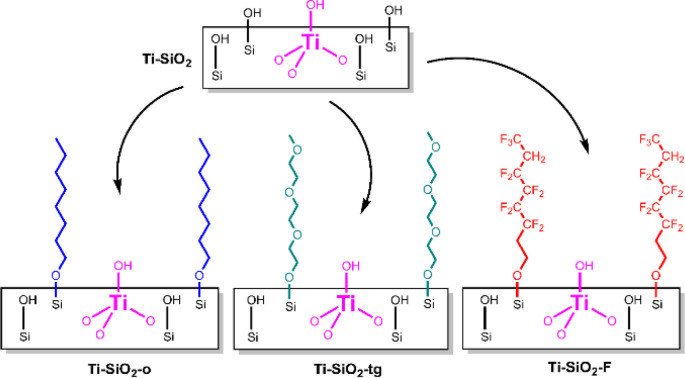

Post-synthetic modification provides an opportunity to tune the surface properties (*e.g.* hydrophilicity/hydrophobicity) of previously-synthesized, supported catalysts. For example, surface modification to remove surface hydroxyls and to increase hydrophobicity of a surface is an effective way to reduce the negative effects of water in liquid-phase selective oxidation chemistry.^1,2^ This increase in hydrophobicity is particularly useful when water adsorbs onto the active metal site competingly with a reactant. Beyond altering surface hydrophilicity/hydrophobicity, other post-synthetic modifications include overcoating of metal oxide layers,^3,4^ grafting of functional organic groups,^5,6^ and depositing additional active metal oxide sites.^7,8^ For liquid phase reactions, any surface modifications must compete against the solvent for any changes to the local environment around the active site. Here, we report grafting of organic molecules to change surface properties for vapor phase cyclohexene epoxidation with vaporized H_2_O_2_, where we hypothesized that surface modification might have a more direct impact on the elementary steps of catalysis. By condensing the corresponding terminal alcohols with surface silanols, we grafted (Scheme 1) three different types of functional groups on a pre-synthesized Ti-SiO_2_ Lewis acid catalyst: octyl groups (Ti-SiO_2_-o, nonpolar), triethylene glycol monomethyl ether (Ti-SiO_2_-tg, polar aprotic), and 1*H*,1*H*-perfluoro-1-octyl (Ti-SiO_2_-F, fluorous).

**Scheme 1:**
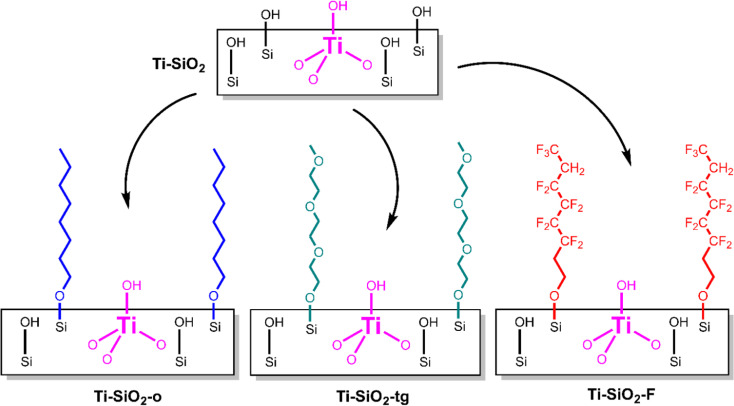
Schematic diagram of grafting of 1-octanol (nonpolar), triethylene glycol monomethyl ether (polar aprotic), and 1*H*,1*H*-perfluoro-1-octanol (polar protic) on Ti-SiO_2_.

We prepared a highly dispersed silica supported Ti catalyst (Ti-SiO_2_) *via* liquid-phase grafting of trichloro(pentamethylcyclopentadienyl)titanium(iv) onto a mesoporous silica support at 0.2 Ti atoms per nm^2^, followed by calcination, which is known to give high specific activity in H_2_O_2_ activation.^9^ We modified the parent Ti-SiO_2_ by grafting the corresponding terminal alcohol in refluxing toluene for 24 h, Soxhlet extraction for 24 h in toluene to remove any ungrafted species,^2^ and drying at 100 °C under vacuum.

Successful grafting is indicated by slight decreases in BET surface area (Fig. 1(a) and Table 1) without changes to the shape of physisorption isotherm and by mass losses in thermogravimetric analysis (Fig. 1(b)). Mass losses beyond the low-temperature desorption of water are due to combustion or decomposition of the grafted species. The water desorption temperatures of modified catalysts are similar, which agree with the previous study.^2^ Mass losses beyond the shaded regime in Fig. 1(b) correspond to loadings of 0.75, 0.42, and 0.30 groups per nm^2^, for Ti-SiO_2_-o, Ti-SiO_2_-tg, and Ti-SiO_2_-F, respectively. Grafting 1-octanol on a Ta-SiO_2_ catalyst was previously reported to give 0.39–0.64 groups per nm^2^.^2^ Most importantly, all these values are higher than the surface Ti loading of parent supported catalyst, which is 0.2 Ti atoms per nm^2^, so that they should be sufficient to affect catalytic behavior of Ti-SiO_2_.

**Fig. 1:**
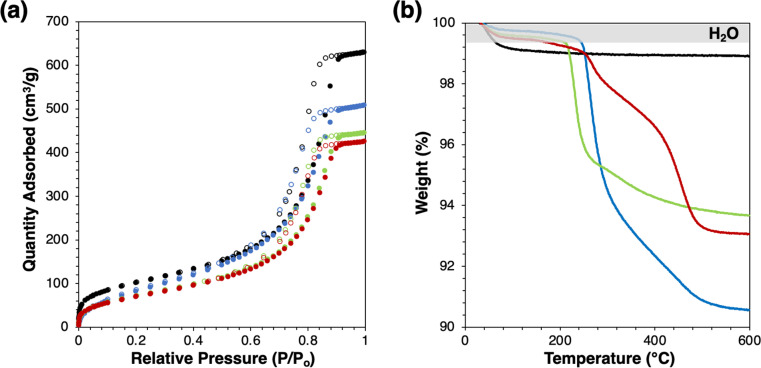
(a) N_2_ physisorption isotherm and (b) mass loss profile by thermogravimetric analysis of catalysts (black: Ti-SiO_2_, blue: Ti-SiO_2_-o, green: Ti-SiO_2_-tg, red: Ti-SiO_2_-F).

**Table 1:** Summary of catalyst properties and activities

Catalyst	BET surface area [m^2^ g^−1^]	Organic surface density [# nm^−2^]	Steady state TOF^a^ [mol_C_6__ mol_Ti_^−1^ h^−1^]	Steady state selectivity^a^ [%] (epoxide/diol/CO_*x*_)
Ti-SiO_2_	370	—	6.5	39/42/19
Ti-SiO_2_-o	340	0.75	15.5	6/86/8
Ti-SiO_2_-tg	270	0.42	11.0	4/85/11
Ti-SiO_2_-F	290	0.30	1.6	18/32/50

a TOF values of production of epoxide and diol values at 600 min. Steady state operation is reached after 200–400 minutes at these conditions.

We performed vapor-phase cyclohexene epoxidation at 120 °C, 3 kPa of cyclohexene, and 3 kPa of vaporized H_2_O_2_ employing our custom built reactor.^10^ Here, we used H_2_O_2_ in acetonitrile, dried over MgSO_4_, to minimize initial water content.^11^ In this study, products were detected with online GC-FID and an in-jet methanizer. We do not observe any C_6_ derived products other than cyclohexene epoxide (epoxide) and *trans*-1,2-cyclohexanediol (diol), consistent with our previous work with Ti-SiO_2_ at similar conditions.^10^ In these systems, cyclohexene first converts to epoxide, and then hydrolyzes to the *trans*-diol (Scheme 2). This stepwise conversion of cyclohexene is consistent with our previous studies,^7,10^ as we do not observe any *cis*-diol that is the product of direct *cis*-dihydroxylation of cyclohexene. Radical oxidation to cyclohexenone or cyclohexenol is not observed. Background over-oxidation to CO and CO_2_ occurs at a rate of approximately 1.2 to 4.1 mol_cyclohexene_ mol_Ti_^−1^ h^−1^ or 0.2 to 0.8% conversion at these conditions, regardless of catalyst.

**Scheme 2:**
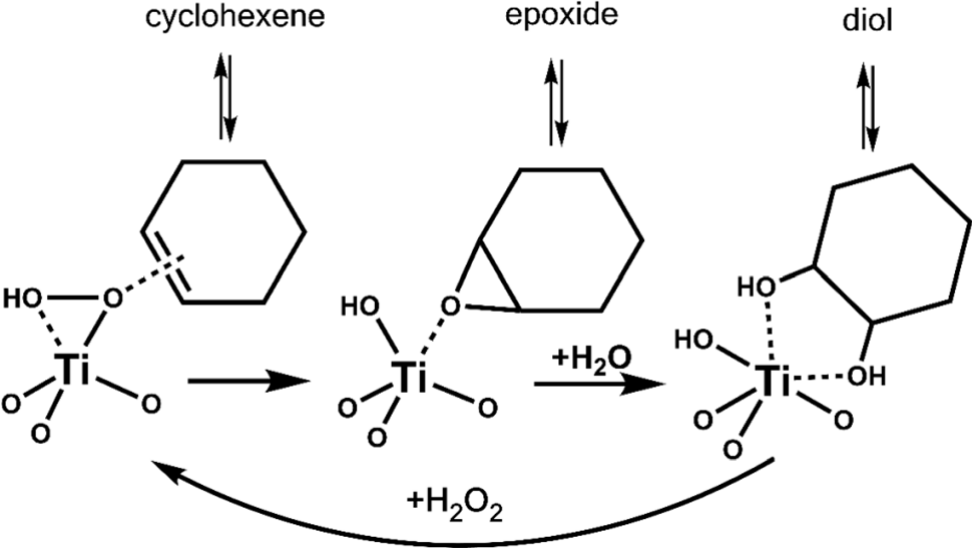
Reaction network of cyclohexene epoxidation to cyclohexene oxide (epoxide), and its hydrolysis to yield *trans*-1,2-cyclohexane diol (diol). Surface modifications near the active site can alter the strength of adsorption of reactants and intermediates, altering product selectivity.

The parent Ti-SiO_2_ shows an initial turnover frequency (TOF = mol_(epoxide+diol)_ mol_Ti_^−1^ hr^−1^) of 19.6 h^−1^ at 50 min time-on-stream (TOS), which decays to a steady-state rate of 6.5 h^−1^ at 600 min TOS. As seen mostly clearly in the selectivity plot, steady-state is reached after ∼200 minutes, with only slow catalyst deactivation thereafter (Fig. 2). The steady state selectivity is 39%/42% to epoxide and diol respectively, with the remainder going to background overoxidation to CO_*x*_. Grafting of a fluorous group (Ti-SiO_2_-F) almost totally suppresses C_6_ product formation. The small amount of remaining C_6_ formation has a selectivity of 18%/32% to epoxide and diol, relatively similar to the parent catalyst and suggesting the existence of small patches of unfunctionalized surface. Otherwise, the conclusion is that the fluorous surface makes binding and activation of cyclohexene unfavorable by inhibiting the adsorption of cyclohexene on the surface. Conversely, grafting of either nonpolar octyl or polar aprotic tri(ethylene glycol) groups on Ti-SiO_2_ increases rates by at least 1.7-fold at steady-state, relative to the parent catalyst. These enhanced catalytic rates have two effects. First, the loss of C_6_ to background overoxidation drops dramatically, from 19% in the parent catalyst to 8–11% in the modified catalysts. Moreover, the C_6_ selectivity shifts to substantially favor hydrolysis of the epoxide to the diol, giving approximately 5%/85% for epoxide and diol, respectively, at steady state.

**Fig. 2:**
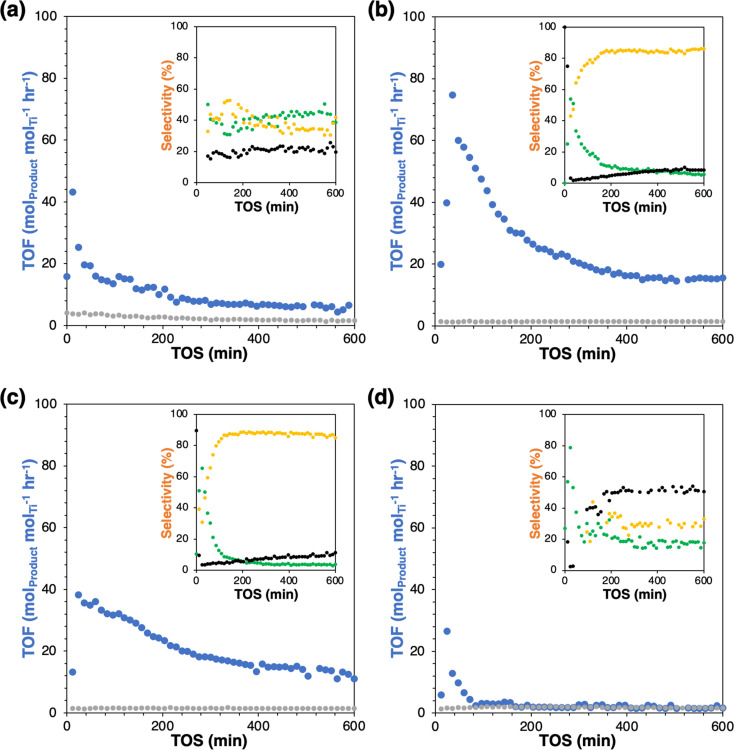
Time-on-stream turnover frequency (TOF) and selectivity (inset) of (a) Ti-SiO_2_, (b) Ti-SiO_2_-o, (c) Ti-SiO_2_-tg and (d) Ti-SiO_2_-F (blue: TOF of C_6_ products, grey: TOF of CO_*x*_/6, green: epoxide selectivity, yellow: diol selectivity, black: CO_*x*_/6 selectivity).

This behavior is quite different from that observed in the condensed phase, where grafting groups to remove surface silanols tends to decrease yields slightly and increase epoxide selectivity relative to diol by suppressing water sorption at the active site.^2^ In the vapor phase and for these wide-pore materials, the lack of a liquid solvent phase means that surface modification can more directly influence the stability of reaction intermediates. As suggested in Scheme 2, the surface modifications appear to be strengthening the adsorption of cyclohexene and the intermediate epoxide, leading to corresponding increases in rate and selectivity to the hydrolysis product. In addition, a recent study by Leonhardt *et al.* proposed computationally that an epoxide molecule can remain adsorbed to one facet of the Ti-OH site while still leaving another coordination site available for oxidation of an incoming cyclohexene.^12^ In that mechanism, enhancing epoxide adsorption at the active site increases hydrolysis to the diol without inhibiting – or even enhancing – overall product formation rates, such as we have observed with the octyl- and tg-modified surfaces. Overall, these observations show that surface grafting can play a significant role in modifying the reactivity of catalysts in the nascent field of selective oxidation with vaporized H_2_O_2_. Also, the results presented here contribute/expand to the current design strategy of post-modification of heterogeneous catalysts with simple method. Additional studies will be carried out to understand the precise mechanistic origins of these changes in rate and selectivity and develop further strategies to tune catalyst surface properties.

## Data availability

The data supporting this article have been included solely in the manuscript.

## Conflicts of interest

The authors declare no competing financial interest.

## Supplementary Material

RA-014-D4RA04552A-s001
