# Supplementary material for: Effect of surface modification on silica supported Ti catalysts for cyclohexene oxidation with vapor-phase hydrogen peroxide
Source: RSC Adv. 2024 Aug 13;14(35):25425–8. doi: 10.1039/d4ra04552a (PMC11321206; doi:10.1039/d4ra04552a)
Supplement: RA-014-D4RA04552A-s001 [file RA-014-D4RA04552A-s001.pdf]

Supplementary Information (SI) for RSC Advances.
This journal is © The Royal Society of Chemistry 2024

[Supplementary Information]

Effect of Surface Modification on Silica Supported Ti Catalysts for Cyclohexene Oxidation with Vapor-phase Hydrogen Peroxide

Sol Ahn,[a*] Sarah K. Friedman,[b] and Justin M. Notestein[*b,c]

[a]Department of Chemical Engineering, Chung-Ang University, 84 Heukseok-ro, Dongjak-gu, Seoul, 06974, Republic of Korea

[b]Department of Chemical and Biological Engineering, and [c]Center for Catalysis and Surface Science, 2145 Sheridan Rd, Evanston, Illinois 60208, United States

Synthesis of Ti-SiO_2

5 g of Selecto SiO_2 (Fisher Chemical, 6 nm pore size, 550 m^2/g, 63−200 μm particle size) was dried for 30 min at 120 °C in static air. Then, the SiO_2 was added to a 250 mL round-bottom flask along with 50 mL of distilled anhydrous toluene (Sigma-Aldrich, ACS Reagent, > 99.5%) and stirred at 200 rpm with a Teflon stir bar at ambient temperature under N_2. The titanium precursor, 264 mg of (pentamethylcyclopentadienyl)titanium(IV) trichloride (Cp*Ti(IV)Cl_3, Strem Chemicals, > 99%) was added to the suspension of SiO_2 in toluene. The suspension was stirred for 1 h at ambient temperature, then vacuum filtered, and washed with 200 mL each of fresh toluene and hexanes (Fisher Chemical, Certified ACS, > 98.5%). The as-synthesized material was loaded into ceramic boats in a muffle furnace and then calcined in static air at 550 °C for 6 h with a 10 °C/min ramp rate to yield Ti−SiO_2, which was used for subsequent grafting of organic molecules.

Modification of Ti-SiO_2

The parent Ti-SiO_2 (~0.5 g) was modified by grafting the corresponding terminal alcohol in refluxing toluene (distilled anhydrous, 20 mL) for 24 h at 140 °C, followed by Soxhlet extraction for 24 h in toluene to remove any ungrafted species, and drying at 100 °C under vacuum. The terminal alcohol was added to solution at levels of 10 mmol/$g_{Ti\text{-}SiO2}$, well in excess of expected or achieved loadings, to ensure modification of the surface. The amounts used of each grafting

molecule are: 0.63 mL of 1-octanol, 0.64 mL of triethylene glycol monomethyl ether, and 1600 mg of 1H,1H-perfluoro-1-octanol.

Characterization of Catalysts

Nitrogen adsorption/desorption isotherms were collected at normal boiling point of liquid N_2 (nominally 77 K) using a Tristar II (Micromeritics) and surface area values were calculated using BET equation. Prior to N_2 physisorption measurements, samples were degassed at 120 °C under dynamic vacuum for 18 h using a Smart VacPrep (Micromeritics). Thermogravimetric analysis (TGA) was performed on a TGA/DCS 1 system (Mettler Toledo AG), which runs on an equipped data processing software (STARe). Catalyst samples were heated from 25 to 600 °C at a ramp rate of 5 °C/min under air (flow rate: 20 mL/min).

Catalysis

Vapor-phase cyclohexene epoxidation tests were performed at 120 °C in a one-to-one ratio of reactant (cyclohexene) and oxidant (H_2O_2). Surface modified catalysts (40 mg) were mixed in 200 mg of quartz sand to minimize possible heat transfer effects, and the catalyst beds were supported on quartz wool in the middle of a quartz tube reactor. Cyclohexene (Sigma-Aldrich, > 99.0%) was introduced by flowing He (5 mL/min, 99.999%, Airgas) through a glass bubbler at ambient temperature, and appropriate amount of He was added to ensure 3 kPa of cyclohexene partial pressure. A solution of 4M H_2O_2 in acetonitrile was loaded in a plastic syringe and injected directly via a PTFE tube into the reactor with a rate of 0.2 mL/h, corresponding to 3 kPa of H_2O_2 partial pressure after it vaporizes. Cyclohexene and C_6 products (cyclohexene oxide, *trans*-1,2-cyclohexanediol) were detected and separated using a gas chromatograph (GC) (Agilent 7890) with an HP-INNOWAX (50 m length, 0.2 mm diameter, 0.4 μm film) column equipped with an in-jet methanizer and flame ionization detector (FID). The following species were not observed above detection limits: benzene, cyclohexane, cyclohexanol, cyclohexanone, cyclohexenol, cyclohexenone, or *cis*-cyclohexanediol.

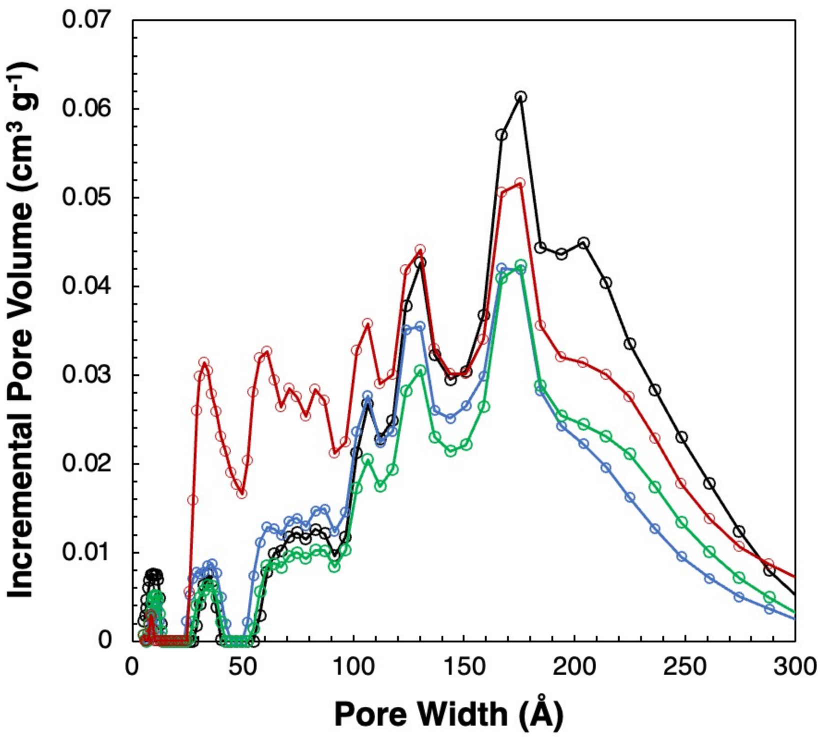

Figure S1. DFT pore size distribution of catalysts

(Black: Ti-SiO_2, blue: **Ti-SiO_2-o**, green: **Ti-SiO_2-tg**, red: **Ti-SiO_2-F**).
